# Single-particle imaging by x-ray free-electron lasers—How many snapshots are needed?

**DOI:** 10.1063/1.5144516

**Published:** 2020-03-20

**Authors:** I. Poudyal, M. Schmidt, P. Schwander

**Affiliations:** Department of Physics, University of Wisconsin-Milwaukee, 3135 N. Maryland Ave., Milwaukee, Wisconsin 53211, USA

## Abstract

X-ray free-electron lasers (XFELs) open the possibility of obtaining diffraction information from a single biological macromolecule. This is because XFELs can generate extremely intense x-ray pulses that are so short that diffraction data can be collected before the sample is destroyed. By collecting a sufficient number of single-particle diffraction patterns, the three-dimensional electron density of a molecule can be reconstructed *ab initio*. The quality of the reconstruction depends largely on the number of patterns collected at the experiment. This paper provides an estimate of the number of diffraction patterns required to reconstruct the electron density at a targeted spatial resolution. This estimate is verified by simulations for realistic x-ray fluences, repetition rates, and experimental conditions available at modern XFELs. Employing the bacterial phytochrome as a model system, we demonstrate that sub-nanometer resolution is within reach.

## INTRODUCTION

X-rays have been used for more than sixty years^1^ to determine the structures of proteins and other biologically important macromolecules. Protein structures are determined by the interpretation of electron density maps obtained from measured structure factors. Since the interaction of x-rays with matter is weak, crystals are widely used to determine these structure factors. When the crystals are exposed to x-ray radiation, diffraction is amplified along specific directions that are determined by Bragg's law. In this way, structure factor amplitudes can be measured with sufficient precision. However, the phase of the structure factors is not experimentally accessible and needs to be retrieved from additional experiments.^2^ X-ray free-electron lasers (XFELs)^3^ can provide enormous incident intensities so that diffraction is sufficiently strong to access structure factor amplitudes from single particles. The particles are destroyed shortly after exposure with the incident radiation. However, XFEL pulses are short enough to collect a diffraction signal before the object is damaged. This is the so-called “diffraction-before-destruction” principle.^4–6^ In a single-particle imaging (SPI) experiment, a large number of two-dimensional (2D) diffraction patterns (snapshots) of single molecules are recorded by a pixel area detector. These snapshots are extremely noisy and taken in random and unknown orientations. Therefore, the orientations of the molecules relative to each other have to be determined from the snapshots. Multiple algorithmic methods of orientation recovery have been developed to assign orientations to single-particle x-ray diffraction patterns.^7–11^ The oriented patterns are merged into a three-dimensional (3D) diffraction volume with phases initially unknown. The phases can be retrieved from the diffraction volume by iterative phasing.^12,13^ Finally, from the phased diffraction volume, the electron density map is determined.

Electron density reconstructions from experimental SPI datasets collected at XFELs have achieved resolutions in the regime of a few tens of nanometers.^14,15^ Diffraction up to a resolution of 5.9 Å was already observed,^16^ but a reconstruction of a 3D diffraction volume was not attempted due to the small number of diffraction patterns. This immediately raises the central question of how many diffraction patterns must be collected for a 3D reconstruction of the electron density for a given resolution. In other words, how many diffraction patterns are required to obtain a diffraction volume at a signal-to-noise ratio (SNR) sufficient to reach the desired resolution by iterative phasing? Clearly, this depends on the molecule under investigation and experimental conditions such as the wavelength, beam size, and incident x-ray fluence. To answer this important question is the primary focus of this paper.

Since the beamtime at XFELs is expensive, and sparsely available, it is important to have a sound estimate of the required number of diffraction patterns to design such an experiment. In contrast to XFELs, electron microscopes are now ubiquitously available. Using cryogenic electron microscopy (cryo-EM), the structures of single molecules have been determined at near-atomic resolution,^17^ which surpasses the resolution reached at XFELs to date. However, the duration of the ultrashort x-ray pulses is faster than the molecular fluctuations. Accordingly, the XFEL provides a snapshot of a molecule “frozen in time” during x-ray exposure. Ambient temperatures are necessary to keep the molecules alive and enable protein dynamics. Using single-particle imaging at the XFEL, such dynamics can be probed with unprecedented time resolution down to the femtosecond regime. In contrast, cryo-EM requires quenching the sample to cryogenic temperatures, which may alter the structure,^18^ and with cryo-EM, the time resolution is limited to a few milliseconds.^19^

**FIG. 1.**
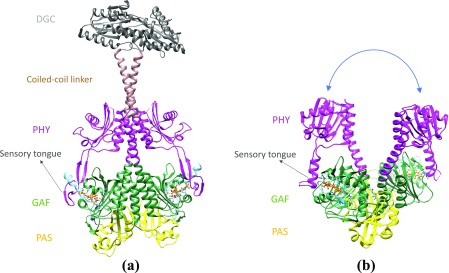
Pr and Pfr structures of *Idiomarina sp*. and *D. radiodurans* phytochrome. (a) Full-length dark-adapted red-light absorbing Pr state^29^ (pdb code 5llw) of the *Idiomarina sp*. phytochrome. Individual domains are colored in yellow, green, magenta, brown, and gray for PAS, GAF, PHY, and coiled-coil and di-guanylyl cyclase (DGC) effector domains, respectively. (b) The photosensory core module (PCM) from the *D. radiodurans* phytochrome in the photoactivated far red-light absorbing Pfr state^28^ (pdb code 5c5k). The PAS, GAF, and PHY domain constitute the PCM and are represented with the same color code as in (a). The PHY domains are displaced substantially in the Pfr structure [blue curved arrow (b)]. The sensory tongue is marked in both structures, and the biliverdin (BV) chromophores (orange) are shown as ball-and-stick models.

So far, the SPI techniques at XFELs were applied to large biological assemblies, primarily viruses.^14,15,20^ Here, we estimate by simulation how the SPI approach could be applied to a much smaller protein at a more relevant, molecular resolution. We selected the phytochrome, a light regulated enzyme, as a suitable model system. Phytochromes are red light photoreceptors characterized in plants, fungi, and bacteria and undergo large structural changes after red light absorption. The full-length, functional bacterial phytochromes (BphPs) consist of multiple domains. The PAS (Period ARNT Sim), GAF (cGMP phosphodiesterase/adenylyl cyclase/FhIA), and PHY (phytochrome-specific) domains form the photosensory core module (PCM).^21–25^ An effector domain has enzymatic activity, which is covalently linked to the PHY domain. The PHY domain has a tongue-like structure, which contacts the GAF domain to seal the biliverdin (BV) chromophore pocket^22,26^ as shown in Fig. 1. Upon photoexcitation, phytochromes interconvert between a dark-adapted red-light absorbing state, Pr, and a photoactivated far red-light absorbing state, Pfr. The sensory tongue probes the configuration of the BV chromophore and transmits the signal to the PHY domain. The structure of the tongue undergoes substantial changes between the Pr and Pfr states.^27,28^ In the Pr state, the tongue assumes a loop to β-strand conformation, whereas in the Pfr state, it assumes a loop to α-helix conformation. Accordingly, large-scale conformational changes with amino acid displacements across several tens of Å between the Pr and Pfr states are required.^28–30^ However, the molecular details of the structural changes during the Pr to Pfr transition and their long-range effects on the effector domains are not well understood. Such changes may not be accommodated by the crystal lattice and thus hidden in crystallographic methods so that SPI is required. With the advancement in x-ray technology, we anticipate that single-particle x-ray experiments on the full-length phytochromes can be conducted at sub-nanometer resolution. With this, the structural dynamics of the Pr to Pfr transition in the full-length functional BphPs could be observed. Here, we estimate how many diffraction patterns are required to determine a 3D diffraction volume for an intact (full-length) phytochrome in the Pr form that can be successfully phased to calculate the three-dimensional electron density map at a targeted resolution.

## METHODS

The simulations reported in this paper were done for the full-length *Idiomarina sp*. phytochrome molecule [protein data bank (PDB) entry 5llw] whose structure was recently determined.^29^ This structure has an approximate diameter *D* of 164 Å and consists of about 11 000 atoms. Diffraction patterns were simulated according to the formalism in Appendix A and implemented in Python. The simulations were done for a photon energy of 2.48 keV corresponding to a wavelength of 5.0 Å. The resolution at the edge of the detector was 10 Å. The phases needed to recover the electron density can be retrieved by sampling the continuous diffraction pattern at sufficiently small intervals in reciprocal space. To retain the phase information, these intervals must be smaller than 1/(2D),^31,32^ i.e., oversampled at least twice with respect to the molecular diameter. This determines the size of the Shannon pixels in the diffraction pattern. For phytochrome, 73×73 detector pixels are required to reach a resolution of 10 Å. The simulated signal for each detector pixel was converted to the expected number of photons for an incident x-ray fluence of $10^{20}$ photons/cm^2^ achievable at an XFEL. The measured photon counts follow Poisson statistics.^33^ Accordingly, diffraction patterns were simulated by adding Poisson noise (“shot noise”) to the calculated diffraction signal.

For the simulation, randomly oriented diffraction patterns were generated using uniform random rotation quaternions as described in Appendix B. The (3D) diffraction volume of the molecule was obtained by orienting the noisy diffraction patterns relative to each other. To retrieve the electron density, the entire diffraction volume (reciprocal space) needs to be covered. Only scattering vectors ending on the Ewald sphere contribute to the diffraction pattern of a molecule in a particular orientation. Accordingly, each diffraction pattern accesses a spherical cap of the diffraction volume centered at the origin of the reciprocal space. Consequently, a large number of diffraction patterns (snapshots) from many different molecular orientations are required to fully sample the reciprocal space. The ensemble of all snapshots is then merged into a diffraction volume using a (cone-gridding) algorithm, which has been previously used for SPI at the XFEL.^34^ The diffraction volume is phased by an iterative phasing algorithm.^13^ As a result, the phases of the structure factors are recovered, and the electron density is determined. The resolution is validated using Fourier Shell Correlation (FSC),^35^ a method now widely accepted in cryo-EM single-particle imaging.

## NUMBER OF SNAPSHOTS

Let D be the particle diameter and d be the aimed resolution. We define a dimensionless quantity *R* = “number of resolution elements” as follows:

$$R=\frac{D}{d}.$$ (1)

The number of Shannon voxels in the outermost shell covered by a single diffraction pattern for oversampling by a factor of two is

$$nV_{\mathrm{snapshot}}=\frac{2\pi \left(1/d\right)}{1/2D}=\frac{4\pi D}{d}=4\pi R.$$ (2)

Accordingly, the number of Shannon voxels in the resolution shell is

$$nV_{\mathrm{shell}}=\frac{4\pi {\left(1/d\right)}^{2}}{{\left(1/2D\right)}^{2}}=\frac{16\pi D^{2}}{d^{2}}=16\pi R^{2}.$$ (3)

The probability of an outermost shell voxel hit by a randomly oriented diffraction pattern is therefore

$$p=\frac{nV_{\mathrm{snapshot}\;}}{nV_{\mathrm{shell}}}=\frac{1}{4R}.$$ (4)

Let $\left\langle n\right\rangle$ denote the mean number of expected photons per Shannon pixel of a diffraction pattern at the resolution shell. As single photons are counted by the detector, the signal follows Poisson statistics, $\mathrm{var}\left(n^{2}\right)=\left\langle n\right\rangle$. Accordingly, the signal-to-noise ratio (SNR) is

$$\mathrm{SNR}=\frac{\left\langle n\right\rangle }{\sqrt{v\mathrm{ar}(n^{2})}}=\frac{\left\langle n\right\rangle }{\sqrt{\left\langle n\right\rangle }}=\sqrt{\left\langle n\right\rangle }.$$ (5)

Due to the weak scattering of x-rays from a single molecule, the SNR of a single diffraction snapshot is way too low for any high-resolution information. It is therefore necessary to obtain information from many snapshots by averaging. The number of times *M* a voxel must be hit by a diffraction pattern in order to reach a desired SNR is

$$M\times \left\langle n\right\rangle ={\mathrm{SNR}}^{2}.$$ (6)

As an example, for $\left\langle n\right\rangle =0.002$ (phytochrome at 10 Å resolution), each voxel must be visited at least 500 times to achieve a SNR of 1.

The probability *P* for a single voxel visited at least *M* times by an ensemble of *nS* snapshots is estimated using the following sum of Binomial distributions:

$$P\left(p,M,nS\right)=1-\sum_{k=0\;}^{M-1\;}\left(\begin{array}{c} nS\\ k\; \end{array}\right)\;p^{k}{\left(1-p\right)}^{nS-k}.$$ (7)

Under the assumption that individual Shannon voxels are visited independently (justification given in Appendix C), the joint probability $\tilde{P}$ to observe all voxels at least *M* times is

$$\tilde{P}\left(p,M,nS,nV_{\mathrm{shell}}\;\right)={P\left(p,M,nS\right)}^{\frac{nV_{\mathrm{shell}}}{2}}.$$ (8)

The factor $\frac{1}{2}$ in front of $nV_{\mathrm{shell}}$ in the equation is due to Friedel's symmetry. Using these relations, we can estimate the total number of diffraction patterns needed to cover the entire diffraction volume at any desired probability $\tilde{P}$. For the special case when *M *=* *1, i.e., at very high signal levels, an estimation of the number of snapshots was proposed previously^14^ and is in agreement with our formulation.

Equation (7) cannot be analytically solved for *nS*. Instead, one can calculate the right-hand side with increasing *nS* until the desired probability *P* is reached. An implementation of an efficient algorithm in Python is listed in Appendix D. However, an analytical formula for the number of snapshots can be obtained by using the de Moivre–Laplace theorem, which approximates the binomial distribution by a Gaussian,

$$\left(\begin{array}{c} nS\\ k\; \end{array}\right)\;p^{k}{\left(1-p\right)}^{nS-k}≃\frac{1}{\sqrt{2\pi p\left(1-p\right)nS}}\;e^{-\frac{{\left(k-p\;nS\right)}^{2}}{2p\left(1-p\right)nS}},$$

$$P\left(p,M,nS\right)=1-\sum_{k=0\;}^{M-1\;}\left(\begin{array}{c} nS\\ k\; \end{array}\right)\;p^{k}{\left(1-p\right)}^{nS-k}=\sum_{k=M\;}^{\infty \;}\left(\begin{array}{c} nS\\ k\; \end{array}\right)\;p^{k}{\left(1-p\right)}^{nS-k}≃\frac{1}{\sqrt{2\pi p\left(1-p\right)nS}}\;\int_{M}^{\infty }e^{-\frac{{\left(k-p\;nS\right)}^{2}}{2p\left(1-p\right)nS}}dk=\frac{1}{2}\;\mathrm{erfc}\left(\frac{M-p\;nS}{\sqrt{p\left(1-p\right)nS}}\right).$$

This approximation allows us to write *nS* explicitly,

$$nS\left(p,M,P\right)={\left(\frac{\sqrt{E{\left(P\right)}^{2}+4pM}-E\left(P\right)}{2p}\right)}^{2},$$

$$\text{where}\;E\left(P\right)={\mathrm{erfc}}^{-1}\left(2P\right)\;\sqrt{p\left(1-p\right)}.$$

Since erfc(0)=1, the probability *P* becomes 0.5 for ${nS}_{c}=\frac{M}{p}$. We call *nS_c_* the characteristic number of snapshots, the number required for a single voxel being visited at least *M* times with probability 50%. Together with Eqs. (4) and (6), this yields

$${nS}_{c}=4RM=\frac{4R\;{\mathrm{SNR}}^{2}}{\left\langle n\right\rangle }.$$ (9)

**FIG. 2.**
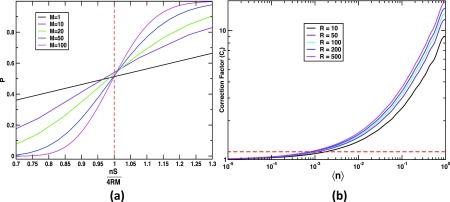
Probability *P* and correction factor *C_f_*. (a) Probability *P* of visiting a single voxel at least *M* times as a function of $\frac{nS}{4RM}$ (*R *=* *16). For the characteristic number of snapshots $nS_{c}=4RM$, all curves admit a probability *P* close to 0.5. (b) Log –log plot of the correction-factor $C_{f}=nS_{e}/nS_{c}$ as a function of $\left\langle n\right\rangle$ for different numbers of resolution elements *R* and SNR = 1.0. The horizontal dashed line corresponds to a value of $C_{f}$ = 1.15. For $\left\langle n\right\rangle <10^{-3},$ the exact number of snapshots $nS_{e}$ is within 15% of the characteristic number $nS_{c}$.

To verify the approximation, we calculated the exact probability P according to Eq. (7) as a function of nS/4RM for different values of *M*. The result is depicted in Fig. 2(a). All curves admit a value of P=0.5 for nS/4RM=1, in close agreement with the approximation (9).

Now, for all voxels being jointly visited at least *M* times, the number of snapshots must be larger of course. With a joint probability $\tilde{P}=0.5\;\mathrm{in}$ Eq. (8), a single-voxel probability $P={\left(1/2\right)}^{2/nV}$ must be reached instead, a value much closer to one. We express the ratio of the exact number of snapshots $nS_{e}$, derived from Eq. (8), to the characteristic number of snapshots from Eq. (9) by a correction factor *C_f_* $=nS_{e}/nS_{c}$. A plot of *C_f_* as a function of the mean number of photons $\left\langle n\right\rangle$ for a SNR value of 1.0 is shown in Fig. 2(b). From this plot, we observe that $nS_{e}$ approaches $nS_{c}$ as the number of photons is lowered. The exact number $nS_{e}$ can easily be estimated from the characteristic number $nS_{c}$ by multiplication with the proper correction factor taken from the graph, without need to solve Eq. (8).

According to Eq. (9), the most important parameter for estimating the number of diffraction patterns is the mean number of expected photons per Shannon pixel $\left\langle n\right\rangle$ at the desired resolution *d*. Different methods to calculate $\left\langle n\right\rangle$ are given in Appendix E. An estimate of the number of snapshots for the full-length phytochrome molecule to reach a SNR of 1.0 at different resolutions and various experimental conditions is tabulated in Table I. The conditions are similar to the experimental conditions available at the existing XFELs such as the Linac Coherent Light Source (LCLS) or the European XFEL (EuXFEL).

**TABLE I.** Estimated number of snapshots required to reach SNR = 1 at different resolutions for the full-length phytochrome molecule at various experimental conditions. $\left\langle n\right\rangle$ is the mean number of photons per Shannon pixel at the desired resolution *d*.

Resolution	Soft x-ray energy = 2.48 keV, λ= 5 Å, beam size = 1.0 μm× 1.0 μm, and fluence =${\;10}^{20}$ ph/cm^2^		Hard x-ray energy = 8.0 keV, λ = 1.5 Å, beam size = 0.1 μm× 0.1 μm, and fluence = ${\;10}^{22}$ ph/cm^2^		Hard x-ray (larger beam) energy = 6.0 keV, λ = 2.07 Å, beam size = 0.5 μm× 0.5 μm, and fluence = $4.0\times 10^{20}$ ph/cm^2^	
	$\left\langle n\right\rangle$	# Snapshots	$\left\langle n\right\rangle$	# Snapshots	$\left\langle n\right\rangle$	# Snapshots
30 Å	1.8 × 10^−2^	1774	1.0 × 10^−1^	488	8.4 × 10^−3^	3394
25 Å	6.7× 10^−2^	5000	4.8 × 10^−2^	1007	3.8 × 10^−3^	8302
10 Å	2.0 × 10^−3^	38 244	2.6 × 10^−2^	4323	2.2 × 10^−3^	35 063
5 Å	^a^	^a^	6.6 × 10^−3^	26 978	5.0 × 10^−4^	284 978
3 Å	^a^	^a^	4.3 × 10^−3^	66 075	3.5 × 10^−4^	672 010

^a^ Not accessible due to the wavelength or unpractically high scattering angle.

## RESULTS

**FIG. 3.**
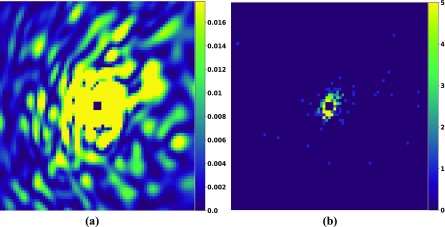
Simulated diffraction patterns. (a) Noise-free diffraction pattern with 10 Å resolution at the edge of the detector and 5 Å wavelength. (b) Diffraction pattern for a photon fluence of $10^{20}$ photons/cm^2^. The total number of scattered photons is 196, and the average photon count ⟨n⟩ at 10 Å resolution is 0.002. The color code corresponds to the number of photons per pixel.

**FIG. 4.**
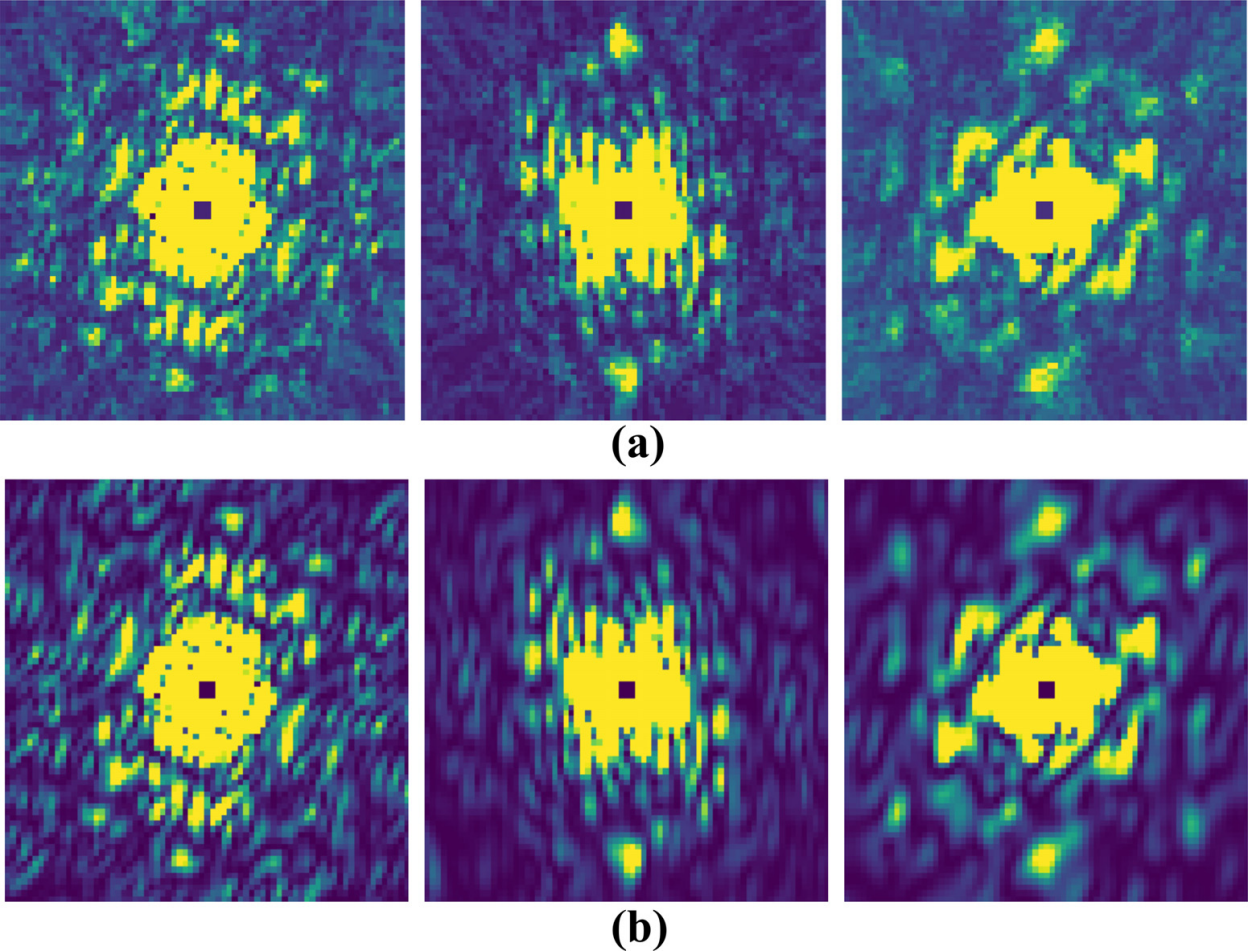
Reconstructed and exact diffraction volume. (a) Central slices of the reconstructed volume, reconstructed from 38 000 noisy patterns. (b) Central slices through the exact, noise-free diffraction volume. The slices correspond to the *xy*, *yz*, and *zx* planes.

**FIG. 5.**
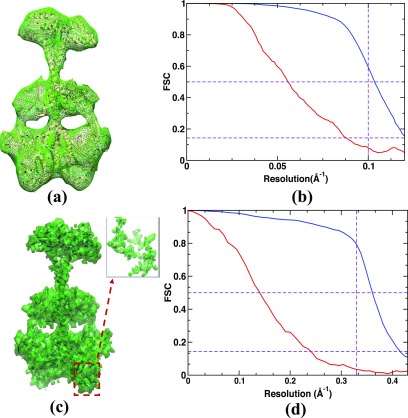
Electron density obtained by iterative phasing and resolution validation by Fourier Shell Correlation (FSC). (a) Reconstructed electron density from 38 000 noisy diffraction patterns of the full-length phytochrome targeted at 10 Å resolution displayed at the 3σ contour level with atomic model superimposed using Chimera.^47^ (b) FSC from (a) for all diffraction patterns (blue) and half the number of patterns (red). (c) Reconstructed electron density from 66 000 noisy diffraction patterns targeted at 3 Å resolution and beam parameters different from (a), as explained in the text. (d) FSC from (c) for all diffraction patterns (blue) and half the number of patterns (red). The horizontal dashed lines show the established thresholds for FSC (0.143 and 0.5). The vertical dashed line corresponds to the target resolution.

Simulated diffraction patterns of the full-length phytochrome are presented in Fig. 3. Figure 3(a) shows a noise-free diffraction pattern, and Fig. 3(b) shows a diffraction pattern corresponding to an incident photon fluence of $10^{20}$ photons/cm^2^ and a photon energy of 2.48 keV (wavelength 5 Å). Only ∼200 photons are scattered from a single phytochrome molecule. Using our formalism [Eq. (8)], 38 000 diffraction snapshots are required to reach a SNR of 1.0 at 10 Å resolution. Accordingly, we simulated noisy 38 000 patterns that were subsequently merged into a 3D diffraction volume. Central slices through the reconstructed 3D volume are shown in Fig. 4(a), while Fig. 4(b) shows a section through the noise-free volume, derived from the atomic model. In the simulation, the central three voxels of the diffraction volume were set to zero, which takes into account the experimentally inaccessible central area of the detectors used at the XFELs. Iterative phasing was used to recover the electron density from the diffraction volume using the combination of the hybrid-input-output (HIO)^12^ algorithm and shrink-wrap algorithm.^13^ The HIO algorithm was applied for the first fifty iterations with feedback parameter β=0.9. After this, the shrink-wrap algorithm was used with an adaptive support constraint determined anew for each iteration cycle. For that, the present electron density was convoluted with a Gaussian of width σ. Voxels that contain electron densities larger than 14% of the maximum were assigned to the new support constraint. The initial width σ was set to six voxels and reduced by 5% after each iteration until a minimum of one voxel was reached. This algorithm converged after a few hundred iterations. The reconstructed electron density at 10 Å is shown in Fig. 5(a).

The resolution and reproducibility of the reconstructed electron density were accessed using Fourier Shell Correlation (FSC). For this, we randomly split the diffraction patterns into two disjoint sets “1” and “2” and processed each set independently, resulting in two electron density maps. The FSC is calculated from the Fourier transformation of the two maps using

$$\mathrm{FSC}\left(q\right)=\frac{\sum_{q}F_{1}\left(q\right)\cdot F_{2}^{*}(q)}{\sqrt{\sum_{q}{\left|F_{1}\left(q\right)\right|}^{2}}\;\sqrt{\sum_{q}{\left|F_{2}\left(q\right)\right|}^{2}\;}},$$ (10)

where $F_{1}\left(q\right)$ and $F_{2}\left(q\right)$ are the Fourier transforms of maps 1 and 2, respectively, q is the magnitude of the scattering vector, and * denotes the complex conjugate. The resolution limit is defined by the value where the FSC drops below a certain threshold. Conventional thresholds used by the cryo-EM community are 0.143 and 0.5.^36,37^ These thresholds are represented by horizontal dashed lines in Fig. 5(b). The FSC drops below 0.5 at around 10 Å [Fig. 5(b), blue line]. This demonstrates that the number of diffraction patterns estimated by Eq. (8), with $\tilde{P}=0.5$ and SNR = 1, is sufficient to reach the targeted resolution. Now, we want to test if a smaller number of snapshots could be sufficient to obtain the electron density at the same resolution. To address this, the reconstruction workflow is repeated with half the number of patterns (19 000), which corresponds to a SNR of 0.67. The FSC [Fig. 5(b), red line] reveals that instead of 10 Å, only about 20 Å is reached in this case.

To evaluate whether near-atomic resolution could be realistically reached by an XFEL experiment, the same pipeline is repeated for a target resolution of 3 Å. For that, a higher photon energy of 8.27 keV is used instead, which corresponds to a wavelength of 1.5 Å. A smaller x-ray focal spot with 100 nm diameter is chosen, which yields a photon fluence of $1\times 10^{22}$ photons/cm^2^. A total of ∼2000 photons/pattern are scattered per phytochrome molecule. According to our formalism, 66 000 noisy patterns are required to reach a resolution of 3 Å. The reconstructed electron density at 3 Å is shown in Fig. 5(c). Details of the structure can be identified from the inset of Fig. 5(c). We also validated the resolution of 3 Å by FSC [Fig. 5(d)]. With half the number of patterns (SNR = 0.65), the resolution reaches only about 8 Å [Fig. 5(d), red line].

**FIG. 6.**
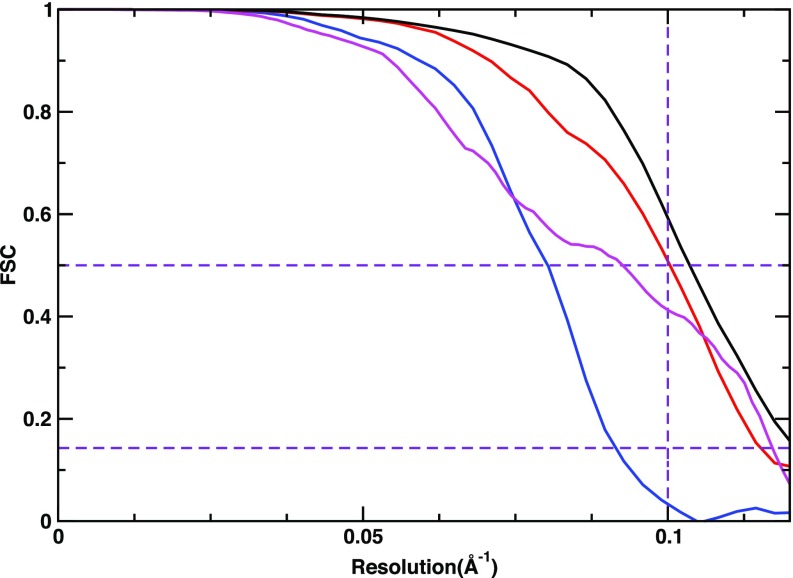
Effect of different types of backgrounds on the resolution. Fourier Shell Correlation (FSC) for a uniform background with the same magnitude as the phytochrome signal at 10 Å (red) and a uniform background three times the magnitude (blue). The FSC of a *q*-dependent background representing a gas of helium with a magnitude equal to the signal at 10 Å (magenta). The FSC without a background is included for comparison (black).

Finally, we analyze the effect of different types of backgrounds on the quality of the reconstruction. For that, we consider a uniform background with the same magnitude as the phytochrome signal at 10 Å and a uniform background three times the magnitude. Additionally, we take a *q*-dependent background assuming a helium gas, using the atomic scattering factors of He. The magnitude of the helium background is set equal to the signal of the phytochrome at 10 Å. The diffraction patterns, including the background, are converted to photon counts by addition of Poisson noise as described above. We repeat the merging and reconstruction processes to obtain 3D electron density maps. The FSC with a background is depicted in Fig. 6 and compared with the FSC without any background. Addition of a uniform background equal to the molecular signal (Fig. 6, red line) and the helium background (magenta line) has a small effect, and the target resolution of 10 Å can still be reached with 38 000 diffraction patterns. For a background three times the molecular signal, however, the resolution is reduced to 12 Å (blue line). We conclude that a background comparable to the molecular signal does not need substantially more diffraction patterns to reach the target resolution.

## SUMMARY

We estimated the appropriate number of snapshots required in order to reconstruct the three-dimensional electron density of a biological molecule at any desired resolution. Quantitative results are derived as a function of the desired resolution, the molecular size, the expected average number of photons per Shannon pixel, and the SNR in the resolution shell. Using this formalism, we demonstrated that a SNR of 1.0 and a joint probability $\tilde{P}=0.50$ are sufficient to reach the desired resolution by iterative phasing validated by Fourier Shell Correlation (FSC). The derivation assumed that orientation recovery does not require additional snapshots. Indeed, a study of single-particle diffraction imaging recently reported successful reconstruction of electron density from diffraction patterns at a signal level of less than 100 photons on the average pattern.^38^ We, therefore, conclude that for a protein like the phytochrome, where 200 photons per diffraction pattern can be expected in an XFEL experiment (see Fig. 3), additional snapshots for orientation recovery are not required.

Our formalism can be extended to incorporate other experimental conditions. In this work, we studied the effect of the uniform background and *q*-dependent background from a gas of helium. However, any form of background or other nuisances such as x-ray streaks and variations in the detector response^39–41^ can be incorporated. By including such signals in the simulations, the impact on the required number of snapshots can be evaluated following the same framework outlined in this paper.

## OUTLOOK

The primary challenge of Single-Particle Imaging (SPI) at XFELs is to reach sub-nanometer resolution to visualize the atomic details of biological macromolecules. Most importantly, a sufficiently large number of single-particle diffraction patterns must be collected during the allocated experimental beamtime. With the new generation of high repetition-rate XFELs, which deliver pulse energies of 10 mJ and higher, improvement in sample delivery technology,^42^ and specialized detectors,^43^ we expect to collect tens of millions of snapshots during a single shift. Combined with noise-robust data analysis algorithms for single-particle detection and orientation recovery,^11,15^ the goal may be reached in the near future. According to our estimates (see Table I), it should be possible to reach sub-nanometer resolution even for smaller proteins with molecular masses similar to that of the phytochrome. However, the main advantage of SPI unfolds in the presence of structural variability, associated with the biological function. Manifold-based machine learning algorithms applied to a large ensemble of single-particle snapshots allow us to reveal the concerted structural changes exercised by the sample.^15^ This enables us to map conformational spectra together with energy landscapes, determine possible functional pathways, and compile 3D molecular movies. Performing single-particle diffraction in time-resolved mode can further advance these promising opportunities for understanding the biological function and bring structural biology to a new level.

The relevance of our formalism is that it establishes a sound mathematical formalism to determine the number of snapshots required to answer a specific biological question at any resolution. These estimates will be useful for beamtime proposals to assert that datasets of sufficient quality can be collected. Similarly, it can help beamline scientists to decide whether enough snapshots can be collected during the allocated beamtime. In this way, the feasibility of single-particle experiments can be judged in an objective way and can be used to guide experiments at new and existing XFELs for a broad class of biological macromolecules.

## Figures and Tables

**FIG. 7.**
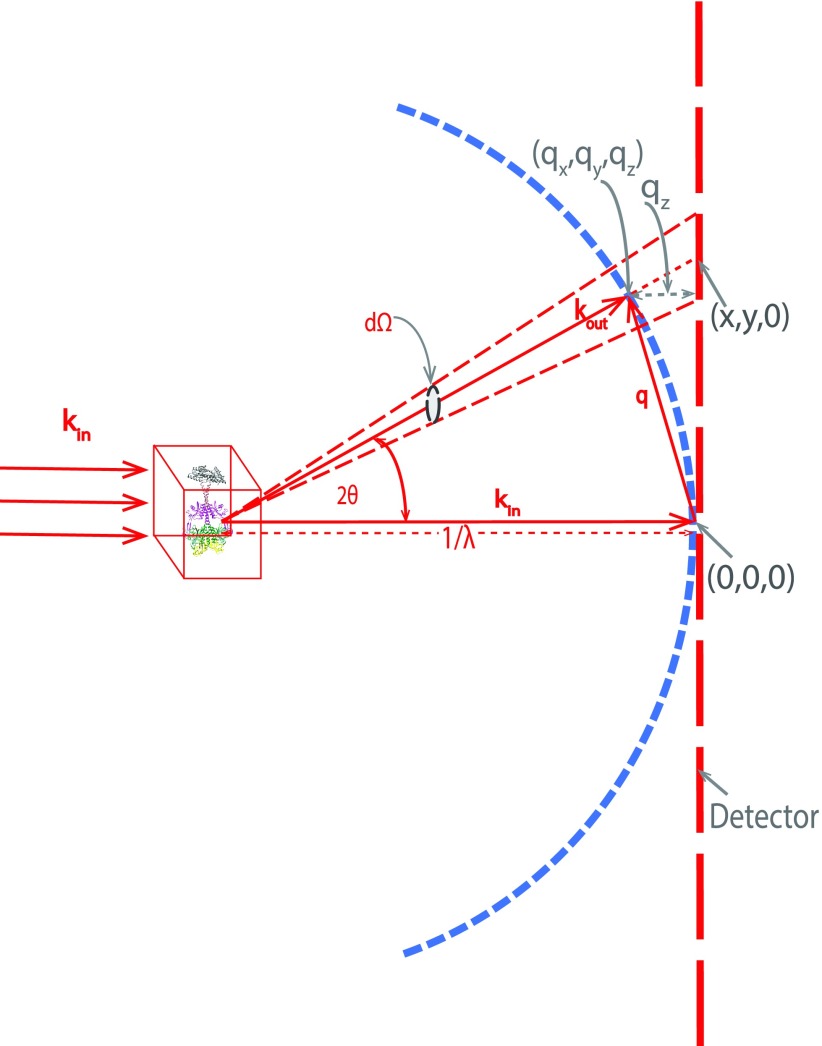
Elastic scattering in forward geometry. 2θ denotes the angle between the incident $k_{in}$ and scattered $k_{\mathrm{out}}$ wave vector. The scattered vector $k_{\mathrm{out}}$ ends on the Ewald sphere due to energy conservation (|$k_{\mathrm{out}}$| = |$k_{in}$|). The scattering vector $q=k_{\mathrm{out}}-k_{in}$ denotes the resulting momentum transfer.

**FIG. 8.**
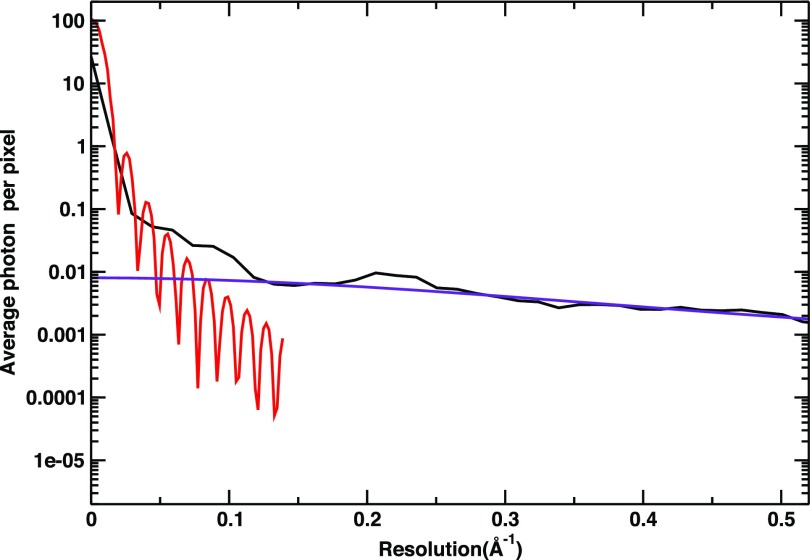
Average number of photons per pixel as a function of resolution. Estimation for the full-length phytochrome using the atomic model (pdb code 5llw) and experimental parameters from Table I, “Hard x-rays” (black). Approximation assuming a spherical molecule filled with carbon atoms (red) and an approximation based on random phases (magenta).

## APPENDIX A: SIMULATION OF DIFFRACTION PATTERNS

For the simulation of diffraction patterns, we construct a regular two-dimensional Cartesian grid, which represents the detector pixel positions. The geometric relation of a detector pixel to the diffraction space is shown in Fig. 7. The coordinates (*x*, *y*) denote the position of a detector pixel, which has a corresponding point (*q*_x_, *q*_y_, *q*_z_) in reciprocal space located on the Ewald sphere, where *q* is the scattering vector. From this geometry, the relationship between the two-dimensional detector and the reciprocal space is given by

$$q=\frac{2}{\lambda }\;\text{sin}\left(\theta \right),$$

$$q_{x}=q\;{\left(1-\frac{\lambda^{2}q^{2}}{4}\right)}^{\frac{1}{2}}\;\text{sin}\left(\arctan2\left(x,y\right)\right),$$

$$q_{y}=q\;{\left(1-\frac{\lambda^{2}q^{2}}{4}\right)}^{\frac{1}{2}}\;\text{cos}\left(\arctan2\left(x,y\right)\right),$$

$$q_{z}=-\frac{\lambda }{2}q^{2}.$$

The expected photon count for a Shannon pixel on the detector with the scattering vector (q) is given by

$$n\left(q\right)=\Phi r_{e}^{2}\;{\left|F\left(q\right)\right|}^{2}d\Omega ,$$

where Φ is the incident flux (“*photons*/*area*” = “*pulse_energy*/*photon_energy*/*area*”), “*o*” is the oversampling ratio, o≥2, and “$r_{e}$” is the classical electron radius $\left(2.8179\times 10^{-5}\;Å\right).\;\;F\left(q\right)$ is the structure factor of the molecule and d Ω the solid angle subtended by the area of the Shannon pixel

$$d\Omega ={\left(\frac{\lambda }{oD}\right)}^{2}{\text{cos}}^{3}(2\theta ).$$

This yields an expected photon count of

$$n\left(q\right)=\Phi r_{e}^{2}\;{\left|F\left(q\right)\right|}^{2}{\left(\frac{\lambda }{oD}\right)}^{2}{\text{cos}}^{3}(2\theta ).$$

For small scattering angles (2θ), the formula can be approximated by

$$n\left(q\right)=\Phi r_{e}^{2}\;{\left|F\left(q\right)\right|}^{2}{\left(\frac{\lambda }{oD}\right)}^{2}.$$

## APPENDIX B: RANDOMLY ORIENTED DIFFRACTION PATTERNS USING QUATERNIONS

To generate uniform random oriented diffraction patterns, we use unit quaternions Q=(a+bi+cj+dk) as follows:^44^

$$Q=(\sqrt{1-u_{1}}\;\text{sin}\left(2\pi u_{2}\right),\sqrt{1-u_{1}}\;\text{cos}\left(2\pi u_{2}\right),\;\sqrt{u_{1}}s\mathrm{in}\left(2\pi u_{3}\right),\sqrt{u_{1}}\;\text{cos}\left(2\pi u_{3}\right)).$$

The three numbers $u_{1},\;u_{2},\;\text{and}\;u_{3}$ are chosen at random, uniformly distributed in the interval $\left[0,\;1\right]$.

These quaternions can be expressed as a rotation matrix^45^

$$R=\left[\begin{array}{ccc} a^{2}+b^{2}-c^{2}-d^{2} & 2bc-2ad & 2bd+2ac\\ 2bc+2ad & a^{2}-b^{2}+c^{2}-d^{2} & 2cd-2ab\;\\ 2bd-2ac\; & 2cd+2ab\; & a^{2}-b^{2}-c^{2}+d^{2} \end{array}\right].$$

This matrix rotates the molecule, represented by the three-dimensional atomic coordinates $\left(x_{j},y_{j},z_{j}\right)$ to a new position with coordinates $\left({x\prime }_{j},\;{y\prime }_{j},\;\;{z\prime }_{j}\right)$, written in matrix form,

$${X\prime }_{j}=RX_{j}.$$

These coordinates are then used to calculate the structure factor of the molecule consisting of *N* atoms, according to

$$F\left(q\right)=\sum_{j=1}^{N}f_{j}\left(q\right)\;\exp(2\pi i\;q\cdot {X^{\prime }}_{j}),$$

where $f_{j}\left(q\right)$ is the atomic scattering factor of atom *j* and *q* is the magnitude of the scattering vector ***q*** (see also Appendix A).

## APPENDIX C: JUSTIFICATION FOR CALCULATING JOINT PROBABILITY

For the calculation of the joint probability $\tilde{P},$ we regarded the statistics for each voxel to be independent of all other voxels, as it is the case for independent dice. However, as a diffraction pattern samples a set of voxels, entirely determined by the orientation of the molecule, the assumption of independence is not granted. To estimate the effect on our formalism, we calculated the joint probability $\tilde{P}$ by simulation as follows: We recorded the actual number of visits for each voxel by merging 4323 diffraction patterns of the phytochrome at 10 Å (see Table I), where the number of patterns was estimated by Eqs. (6) and (8), based on independent voxels with *M *=* *39. Of a total of 27 trials, we found 15 instances with all voxels visited at least *M* times. This corresponds to a probability of 0.55. As the statistical error expected from the number of trials is about ±0.1, this estimation is within the value predicted by the assumption of independent voxels. We therefore conclude that our calculation of the joint probability, as given by Eq. (8), is sufficient for the purpose of the present work.

## APPENDIX D: PYTHON CODE TO ESTIMATE THE EXACT NUMBER OF SNAPSHOTS

**Table:**

import numpy as np
def logFactorial(n):
if n < 20:
value = np.log(np.math.factorial(n))
else:
value = 0.5*np.log(2*np.pi*n) + n*np.log(n/np.e)
return value
def pnm(p,N,M):
#this function gives the probability to observe a voxel at least M times
#from an ensemble of N snapshots
# p = probability to hit a voxel for a single snapshot
# N = Number of Snapshots
# M = Redundancy
if N < M:
s = 1
else:
s = 0
lp = np.log(p)
lmp = np.log(1-p)
for k in np.arange(M):
s = s + np.exp(logFactorial(N) - logFactorial(N-k) - logFactorial(k) + k*lp + (N-k)*lmp)
return np.maximum(1-s,0)
def numberOfSnapShots(d,D,nPhotons,SNR,P_tilde):
#nS number of Snapshots
#d is the Resolution
# D is the Diameter of a Molecule
#nPhotons is the Number of Photons
#P_tilde is the combined probability
#Number of Resolution elements
R = D/d
#number of voxels at Resolution Shell
nV_Shell = 16*np.pi*R**2
#probability per Shannon voxel
p = 1./(4*R)
M = np.ceil(SNR**2/nPhotons)
# P -> Probability to observe a voxel at least M times from an ensemble of nS snapshot
# obtained from given P_tilde
P = np.exp(2*np.log(P_tilde)/nV_Shell)
nSmax = 1e12#
step = 2**10#
nS0 = M
while step > 1:
for nS in np.arange(nS0,nSmax,step):
if pnm(p,nS,M) > P:
break
nS0 = nS - step
step = step /2
return nS

## APPENDIX E: ESTIMATING THE AVERAGE NUMBER OF PHOTONS IN THE OUTERMOST SHANNON PIXELS

The average number of photons $\left\langle n\right\rangle$ can be calculated from the molecular structure factor $F\left(q\right)$ as described in Appendix A. However, this requires calculations based on an atomic model, usually not available. Fortunately, there are useful approximations that only require the molecular weight and the approximate size of the molecule. Representing all non-hydrogen atoms by carbon atoms, the number of atoms $N_{c}$ can be estimated from the molecular weight MW,

$$N_{c}=MW/12\;\mathrm{Da}.$$

At low resolution, one can approximate the molecule by a sphere with diameter *D.*^46^ The squared structure factor of a sphere filled with $N_{c}$ carbon atoms of atomic number $Z_{c}=6$ is

$${\left|F\left(q\right)\right|}^{2}=N_{c}^{2}Z_{C}^{2}{\left(3\frac{\text{sin}\left(\pi qD\right)-\pi qD\text{cos}\left(\pi qD\right)}{{\left(\pi qD\right)}^{3}}\right)}^{2}.$$

Accordingly, the average number of photons becomes

$$\left\langle n\right\rangle =\phi r_{e}^{2}N_{c}^{2}Z_{C}^{2}{\left(3\frac{\text{sin}\left(\pi qD\right)-\pi qD\;\text{cos}\left(\pi qD\right)}{{\left(\pi qD\right)}^{3}}\right)}^{2}d\Omega .$$

With the number of resolution elements R=D/d and $d=\frac{1}{q},$ this can also be written as

$$\left\langle n\right\rangle =\phi r_{e}^{2}N_{c}^{2}Z_{C}^{2}{\left(3\frac{\text{sin}\left(\pi R\right)-\pi R\;\text{cos}\left(\pi R\right)}{{\left(\pi R\right)}^{3}}\right)}^{2}d\Omega .$$

Hence, for the low-resolution approximation, the geometric part of the structure factor depends only on the number of resolution elements.

For the high-resolution regime, a different approximation is quite useful. With $N_{c}$ carbon atoms, the squared structure factor is

$${\left|F\left(q\right)\right|}^{2}={\left|f_{C}\left(q\right)\sum_{j=1}^{N_{C}}\exp(2\pi i\;q{\cdot X}_{j})\right|}^{2}={\left|f_{C}\left(q\right)\right|}^{2}{\left|\sum_{j=1}^{N_{C}}\exp(2\pi i\;q{\cdot X}_{j})\right|}^{2},$$

where $f_{C}\left(q\right)$ denotes the atomic scattering factor of carbon. Taking the average over a shell with radius *q* yields

$$\left\langle {\left|F\left(q\right)\right|}^{2}\right\rangle ={\left|f_{C}\left(q\right)\right|}^{2}\left\langle {\left|\sum_{j=1}^{N_{C}}\exp(2\pi i\;q{\cdot X}_{j})\right|}^{2}\right\rangle .$$

If the complex number $\exp(2\pi i\;q{\cdot X}_{j})$ can be approximated by a random phasor, the quantity $\langle {\left|\sum_{j=1}^{N_{C}}\exp(2\pi i\;q{\cdot X}_{j})\right|}^{2}\rangle$ becomes equal to $N_{C}$^33^ so that

$$\left\langle n\right\rangle =\phi r_{e}^{2}N_{c}{\left|f_{c}\left(q\right)\right|}^{2}d\Omega .$$

For this, high-resolution approximation $\left\langle n\right\rangle$ is directly proportional to $N_{c}$ and ${\left|f_{c}\left(q\right)\right|}^{2}$.

Both approximations are compared with the exact calculation using the atomic model of the full-length phytochrome (pdb code 5llw) and experimental parameters from Table I “Hard x-rays.” As shown in Fig. 8, the low-resolution approximation is reasonable up to a resolution of ∼20 Å, whereas the high-resolution approximation is in excellent agreement for sub-nanometer resolution and below.

## APPENDIX F: COMPUTATIONAL RESOURCES

The computational workflow was implemented with custom-written Python programs. All calculations were performed on a Linux workstation with AMD Processor FX-8350 (Eight-Core) and 16 GB RAM. Computational times were approximately as follows: 6 h for simulation of 30 000 diffraction patterns and 1 h to merge 30 000 diffraction patterns to a 3D diffraction volume using the cone-gridding algorithm.
